# *Catharanthus roseus* (L.) G. Don counteracts the ampicillin resistance in multiple antibiotic-resistant *Staphylococcus aureus* by downregulation of PBP2a synthesis

**DOI:** 10.1515/biol-2022-0718

**Published:** 2023-09-26

**Authors:** Aparna Shil, Sushmit Mukherjee, Prerona Biswas, Sudipta Majhi, Sima Sikdar, Biswadev Bishayi, Mausumi Sikdar née Bhakta

**Affiliations:** Microbiology, Nutrition and Dietetics Laboratory, Physiology Unit, Department of Life Sciences, Presidency University, Kolkata 700073, India; Department of Physiology, Immunology Laboratory, University of Calcutta, University Colleges of Science and Technology, Kolkata 700009, India

**Keywords:** *Staphylococcus aureus*, ethanol extract of *Catharanthus roseus* root, ampicillin resistance, penicillin-binding protein 2a, comet assay

## Abstract

It is essential to revisit the global biodiversity, search for ethnopharmacologically relevant plants, and unveil their untapped potential to overcome the complications associated while treating infections triggered by multiple antibiotic-resistant *Staphylococcus aureus*. *Catharanthus roseus* (L.) G. Don of the Apocynaceae family is a medicinal plant used for remedial purposes against infectious diseases from ancient times. In this study, we intended to evaluate the mechanism by which the ethanolic extract of *C*. *roseus* root (EECRR) causes the reversal of ampicillin resistance in *S. aureus*. To achieve this goal, we have stained EECRR-treated *S. aureus* with acridine orange, analysed DNA damage by comet assay, and studied the alteration of plasmid band pattern and expression of penicillin-binding protein 2a (PBP2a) protein. Experiments revealed better *S. aureus* killing efficiency of EECRR at its minimum inhibitory concentration (MIC) doses due to DNA damage and reducing plasmid band intensities along with a decline in the expression of PBP2a in EECRR-treated cells at half-MIC dose. EECRR proved to be an efficient growth inhibitor of *S. aureus* that reduces the expression of PBP2a. Therefore, EECRR can also render ampicillin-resistant *S. aureus* susceptible to the antibiotic.

## Graphical abstract

**Figure:**
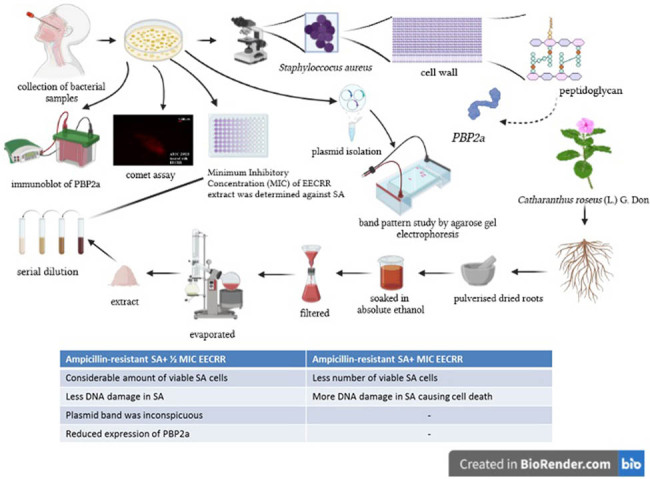

## 1 Introduction

*Staphylococcus aureus*, a well-known commensal microbe found in human skin and mucous membranes, is known to cause bacterial infections upon gaining an opportunity to invade these physical barriers. According to a case study report by Cai et al., a patient with atypical hip pain had developed systemic sepsis due to an infection caused by *S. aureus* and ultimately died of multiple organ failure [1]. It can also colonize in urinary stents and cause urinary tract infection (UTI) in renal transplant patients [2]. Penicillin was initially used to treat these infections, which was replaced by methicillin due to the appearance of penicillin-resistant mutants, as was primarily reported in 1946 [3]. Eventually, methicillin-resistant *S. aureus* (MRSA) appeared in 1961, and the treatment of MRSA infection became more difficult gradually due to its multidrug-resistant properties. One of the reasons behind the emergence of antibiotic-resistant mutants of *S. aureus* is the improper use of antibiotics [4]. Acquisition of the *mecA* gene in MRSA enables them to exhibit resistance against β-lactam antibiotics. Penicillin-binding protein 2a (PBP2a), the product of the *mecA* gene, confers a lower affinity for all β-lactam antibiotics and restricts their therapeutic use [5]. Vancomycin and teicoplanin have reduced effectiveness on many MRSA strains since they have acquired resistance against those drugs [6]. New drugs, such as linezolid, daptomycin, dalfopristin, fidaxomicin, and tigecycline, are used to treat the infection as an alternative to those antibiotics. However, the dynamic changes in the susceptibilities of *S. aureus* to these antibiotics have also been reported [7]. The number of currently approved drugs is insufficient to meet the needs of the therapeutic world, and therefore, the necessity of new antibiotics is on the rise.

Researchers now focus on finding alternative therapeutic agents from natural compounds and restrict the use of synthetic products due to their adverse effects on the host system [8]. Indian biodiversity has flourished with medicinally important plants, several of which have been used as traditional healing remedies since ancient times [9]. One such kind of ethnopharmacologically relevant plant is *Catharanthus roseus* (L.) G. Don (CR) that belongs to the Apocynaceae family, which has been used as a folk medicine for treating fatal diseases [10]. Previously published reports suggest that *C*. *roseus* has antioxidant, antidiabetic, anticancer, anti-helminthic, antimicrobial, hypotensive, and wound-healing properties [10,11]. Moreover, scientific evidence also supports that root and leaf extracts of *C*. *roseus* have antibacterial effects against many pathogenic strains [12,13]. Recently, in our previous studies, we detected the presence of various bioactive phytoconstituents in *C*. *roseus*, such as fatty acids and essential oils that have an antibacterial efficacy [13], and uncovered the mechanism of their antibacterial action against *S*. *aureus* in a rat model [14]. Even though there are only a handful of studies, including our previous work, on *C*. *roseus* fighting against resistant strains of *S. aureus* and *Candida albicans* [15], the mechanism by which *C*. *roseus* effectively render *S. aureus* vulnerable to ampicillin has not been elucidated to date.

Despite the recent trend of using natural resources to manage infections, microorganisms have also developed strategies to overcome bactericidal or bacteriostatic effects of natural product-based drugs [16]. Given the gravity of the situation, our objective was to address bacterial antibiotic resistance and explore strategies to reuse and enhance the effectiveness of currently ineffective antibiotics against multidrug-resistant bacteria. The resistant bacteria were subjected to an herbal extract treatment, which targets to reduce their antibiotic resistance property.

## 2 Materials and methods

### 2.1 Reagents and chemicals

Mueller–Hinton agar, Mueller–Hinton broth (MHB), and ampicillin discs were purchased from HiMedia (Mumbai, India). Tris–HCl, Tris base, ethylenediaminetetraacetic acid (EDTA), agarose, sucrose, 1 kb DNA ladder, bovine serum albumin (BSA), tris buffered saline (TBS)–Tween-20, and Triton-X 100 were purchased from SRL (India). Sodium chloride, disodium hydrogen phosphate, di-hydrogen sodium phosphate, methanol, ethanol, acetic acid, sodium hydroxide, sodium dodecyl sulphate (SDS), and sodium acetate were purchased from E Merck (India), and water was deionized using Millipore Milli-Q system (Bedford, MA). Acridine orange (AO), PBP2a monoclonal antibody, lysostaphin, and proteinase K were purchased from Sigma-Aldrich (St. Louis, MO, USA). An anti-GAPDH monoclonal antibody was purchased from Thermo Fisher Scientific (Waltham, MA, USA). Nitro blue tetrazolium (NBT) and 5-bromo-4-chloro-3-indolyl phosphate (BCIP) were purchased from Promega (Madison, WI, USA). Ethylene glycol tetraacetic acid (EGTA) was purchased from Sigma-Aldrich (St. Louis, MO, USA). Polyvinylidene fluoride (PVDF) was purchased from Merck-Millipore (Bengaluru, India).

### 2.2 Preparation of plant extract

*C. roseus* plants were obtained from the garden maintained by Presidency University, Kolkata, India. The *C. roseus* plant samples were subsequently identified and authenticated by the Botanical Survey of India (No: CNH/57/2013/Tech II/1088, 4th October 2013). For the preparation of ethanol extract of *C*. *roseus* root (EECRR), a previously published method with a slight modification was followed [14]. The modified step is that the dried root powder was soaked in 100 mL of ethanol for 72 h instead of 48 h for better extraction of components.

### 2.3 Bacterial strains

In this study, multiple antibiotic-resistant bacterial strains, namely, PU-CA-14 and MC-CL-10, belonging to the category of community-associated and clinical *S. aureus* strains, respectively, were used. The data regarding their collection and confirmation have been described in our previous studies [13,14]. *S. aureus* ATCC 25923 was used as a standard reference strain.

### 2.4 Antibiotic resistance pattern (ARP) of EECRR-treated bacterial cells

The Kirby–Bauer disc diffusion method [17] was used to determine the ARP of bacterial cells. The cells were grown overnight in the presence of half of the minimum inhibitory concentration (MIC) dose of EECRR (192.4, 48.4, and 196.4 µg/mL for PU-CA-14, *S. aureus* ATCC 25923, and MC-CL-10, respectively, as determined in our previous studies [13,14]). The turbidity of this overnight culture was adjusted by diluting with MHB to make it equivalent to 0.5 McFarland standard so that the culture contains approximately 1.5 × 10^8^ CFU/mL of bacterial cells. Then, 100 µL of such culture was spread over a sterile nutrient agar plate and allowed to stand for 30 min, and commercially available ampicillin discs (10 µg/disc, HiMedia, India) were placed aseptically at the centre of the plate and were incubated at 37°C for 24 h. For the maintenance of proper testing conditions and interpretation of the result, clinical and laboratory standards institute guideline was followed [18].

### 2.5 Determination of MIC of ampicillin by micro-broth dilution assay

MIC values of ampicillin against EECRR-treated multiple antibiotic-resistant *S. aureus* (MAR-SA) strains were determined by the micro-broth dilution assay method [13]. Briefly, 0.05 mL of cell suspension from overnight bacterial culture, having 10^6^ CFU/mL colony count, was added to the wells of a microtiter plate containing serially diluted test solutions of ampicillin, starting from a stock concentration of 1 mg/mL. The microtiter plate was incubated at 37°C overnight. Then, the optical density was recorded spectrophotometrically at 600 nm using a multi-mode reader (Biotek Synergy H1, Vermont, USA) to determine MIC values.

### 2.6 Detection of live and dead bacterial cells by staining with AO

Bacteria were grown in the presence of EECRR, i.e., bacteria were allowed to grow in nutrient broth containing MIC doses of EECRR extract (384.8, 96.8, and 392.8 µg/mL of EECRR for PU-CA-14, *S. aureus* ATCC 25923, and MC-CL-10 respectively), as determined in our previous studies [13,14] for one set and 1/2 MIC doses of EECRR (192.4, 48.4, and 196.4 µg/mL of EECRR for PU-CA-14, *S. aureus* ATCC 25923, and MC-CL-10, respectively) for another set of experiments. Cells were washed and resuspended in 0.1 M phosphate-buffered saline (PBS) and spread over clean grease-free slides to form a thin smear after its density was adjusted to be equivalent to 0.5 McFarland standard (1.5 × 10^8^ CFU/mL). The smears were then fixed in methanol/acetic acid (3:1) solution at 4°C for 14 h and then stained with AO solution (0.19% of AO in 0.1 M phosphate citrate buffer, pH 2.5) for 10 min in the dark. The stained slides were washed with distilled water for 5 min, air-dried, and observed under a fluorescence microscope (Zeiss Axioscope A1, Thornwood, NY, USA) at 1,000× magnification using immersion oil [19]. The excitation filter used was 470 nm, and the barrier filter used was 540–580 nm. The bacterial cells that fluoresce green (maximum emission wavelength: 525 nm) were considered viable, and those that fluoresce red (emission wavelength: ≥630 nm) were considered non-viable due to the abundant damaged DNA. The number of viable cells, the number of dead cells, and the intensity of green and red fluorescence emitted by AO were assessed by ImageJ (NIH, USA; Version 1.52a) software.

### 2.7 Isolation and electrophoresis of plasmid DNA from EECRR-treated bacteria

Two sets of 1.5 mL bacterial cultures of PU-CA-14, *S. aureus* ATCC 25923, and MC-CL-10 strains were grown overnight to extract plasmid DNA. One set was grown in the presence of a 1⁄2 MIC dose of EECRR (192.4, 48.4, and 196.4 µg/mL, respectively), as determined by our previous studies [13,14]. The other set was grown without EECRR, which served as the control for this experiment. Subsequently, the cell pellets were collected, exposed to 2 μL of lysostaphin solution (1.0 μg/mL), and incubated at 37°C for 30 min. Then, 200 μL of an alkaline detergent solution (0.2 N NaOH and 1% SDS) was added, and 150 μL of 3 M sodium acetate (pH 4.8) was introduced. The resulting mixture was then maintained on ice for 10 min.

After centrifugation (Eppendorf, 5424 R, Hamburg. Germany) at 15,000 rpm for 5 min, the supernatant was collected and 1 mL of 95% ice-cold ethanol was added to it and kept at −20°C for 10 min. The supernatant was discarded after centrifugation, and the sediment was resuspended in 50 μL of Tris/EDTA (10 mM Tris–HCl and 1 mM EDTA) pH 8.0. Then, electrophoresis was carried out in 0.5% agarose gel at 30 mA (90 V), and the DNA band was visualized under the ChemiDoc imaging system (BioRad) [20].

### 2.8 Replica plating

To obtain isolated colonies, overnight bacterial culture (1.5 × 10^8^ CFU/mL) of PU-CA-14, *S. aureus* ATCC 25923, and MC-CL-10 was spread on nutrient agar (NA) plates. For preparing the master plate, the isolated colonies were again streaked on fresh NA plates by the streak plate method, which were then replica-plated on NA containing antibiotics (ampicillin, 130 µg/mL), to which the strain is resistant. Then, the cells were treated with a 1/2 MIC dose of EECRR, and replica plating was performed again. Colonies that grew on master plates but failed to grow on plates containing antibiotics were considered plasmid-cured derivatives [21].

### 2.9 Extraction of protein and Western blot analysis

For extracting protein from control and EECRR-treated cells (with 1/2 MIC dose), bacterial cells were harvested from 500 mL of culture, suspended in 4 mL of lysis buffer (0.25 M sucrose, 20 mM Tris–HCl, 2 mM EDTA, and 2 mM EGTA, pH 7.5), and sonicated three times for 30 s at 100 µA in an ultrasonicator (Hielscher UP100H, Teltow, Germany) at an interval of 30 s in between. The supernatant was collected after centrifugation at 8,000×*g* for 10 min, and the protein concentration was measured by the Lowry method [22]. Separation of protein was performed on 10% sodium dodecyl sulphate-polyacrylamide gel electrophoresis with a marker (Precision Plus Dual Color Protein standard, California, USA). Then, the bands were transferred electrically to PVDF (Millipore) membranes and blocked with 5% BSA in TBS–Tween 20 at room temperature for 30 min. Blots were incubated with MRSA anti-PBP2a (1:1,000) monoclonal antibody (Sigma, Aldrich) and anti-GAPDH (1:1,000) monoclonal antibody (Invitrogen, GA1R) at 4°C overnight consecutively. After washing, the blots were again incubated with alkaline phosphatase-conjugated anti-mouse immunoglobulin G secondary antibody (1:2,000) for 1 h at room temperature. Immunoreactive proteins present in the membrane were detected by staining it with a substrate solution of NBT–BCIP. Band intensities were measured using ImageJ (NIH, USA; Version 1.52a) software, and densitometric analysis was carried out using MS Excel and Statsdirect. The significance level so obtained is based on Mann–Whitney *U* multiple comparison test (*p < 0.05, **p < 0.01, ***p < 0.001).

### 2.10 Evaluation of DNA integrity by comet assay

This single-cell gel electrophoresis experiment was carried out following the protocol of Solanky and Haydel [23] with some modifications. Briefly, for preparing the microgel, 100 µL of 5% agarose in PBS was spread over the slide. Aliquot of 10 µL of bacterial culture (grown in the presence of MIC doses that were 384.8, 96.8, and 392.8 µg/mL of EECRR for PU-CA-14, ATCC 25923, and MC-CL-10, respectively, determined in our previous studies [13,14]) containing 1.5 × 10^8^ CFU/mL cells was mixed with 50 µL of 0.5% agarose (low melting) in PBS, spread uniformly over the agarose, and kept it at 4°C for solidification. Then, the microgel was immersed in lysis solution (NaCl, l2.5 M; EDTA, 100 mM; Triton X-100, 1% (v/v); Tris 10 mM, pH 10), supplemented with lysostaphin (1 µg/mL in PBS) for 1 h followed by dipping it into the enzyme digestion solution (NaCl, 2.5 M; Tris, 10 mM; pH 7.4; EDTA, 10 mM) containing 1 mg/mL of proteinase K for 1 h at 37°C. It was placed in an electrophoresis unit flooded with an electrophoretic buffer (NaOH, 300 mM; EDTA, 1 mM; pH 13) and allowed to stand for 20 min to permit the unwinding of damaged DNA under the buffer. Then, it was exposed to neutralizing buffer (0.4 M Tris, pH 7.5) for 30 min after the continuation of electrophoresis in the electrophoretic buffer for 25 min at 25 V. To visualize DNA, the microgel was stained with ethidium bromide (100 µg/mL) for 10 min and focused under a fluorescence microscope (Zeiss Axioscope A1, Thornwood, NY, USA) with fluorescein isothiocyanate filter combination (excitation: 490 nm, dichroic: 500 nm and emission: 510 nm). The degree of DNA damage was calculated in percentage using the CASP software (http://www.casp.of.pl) [24]. The significance level is based on Mann–Whitney *U* multiple comparison test (*p < 0.05, **p < 0.01, ***p < 0.001).

### 2.11 Statistical analysis

The significance levels, based on Mann–Whitney *U* multiple comparison tests, were used for all analyses using Statsdirect 3.0 software. Data were expressed as mean ± standard error (SE), and *p* values less than 0.05 and 0.01 were considered significant.

## 3 Results

### 3.1 Effect of ampicillin on EECRR-treated bacterial cells

**Figure 1:**
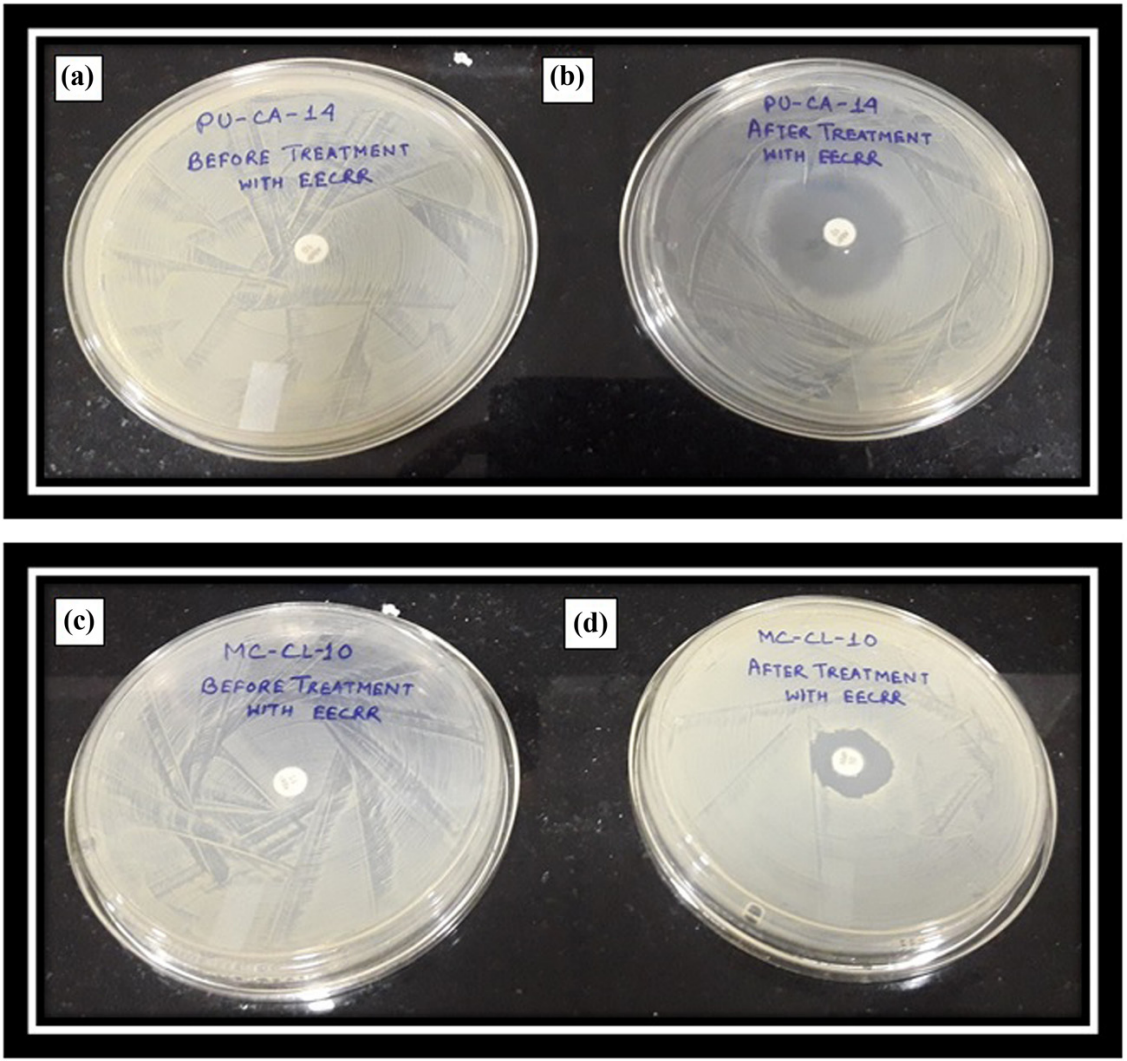
Disc diffusion test with ampicillin (10 µg/disc). (a) PU-CA-14 (control) before treatment with EECRR; (b) PU-CA-14 after treatment with EECRR; (c) MC-CL-10 (control) before treatment with EECRR; and (d) MC-CL-10 after treatment with EECRR.

Around the ampicillin disc, a clear bacterial growth-free region was detected with measurements of 17 and 28 mm for EECRR-treated *S. aureus* strains MC-CL-10 and PU-CA-14, respectively (Figure 1), whereas control cells were resistant to this antibiotic [9,10]. MIC values of ampicillin were noted as 15.5 and 34 µg/mL for EECRR-treated PU-CA-14 and MC-CL-10 cells, respectively (Table 1). There were 8-and 4-fold reductions in MIC values of ampicillin for respective bacterial cells as MICs of control cells were 124.2 and 136.75 µg/mL [13,14].

**Table 1:** MIC values and zones of inhibition of ampicillin against bacterial strains, both before and after treatment with EECRR, respectively

Bacterial strains	MIC (µg/mL)		Zone of inhibition (mm)	
	Before treatment with EECRR	After treatment with EECRR	Before treatment with EECRR	After treatment with EECRR
PU-CA-14	124.2 ± 0.56 [12]	15.5 ± 0.42	0.0	28.0 ± 0.04
MC-CL-10	136.75 ± 0.52 [12]	34 ± 0.37	0.0	17.0 ± 0.02

### 3.2 Impact of AO staining on EECRR-treated bacterial cells

**Figure 2:**
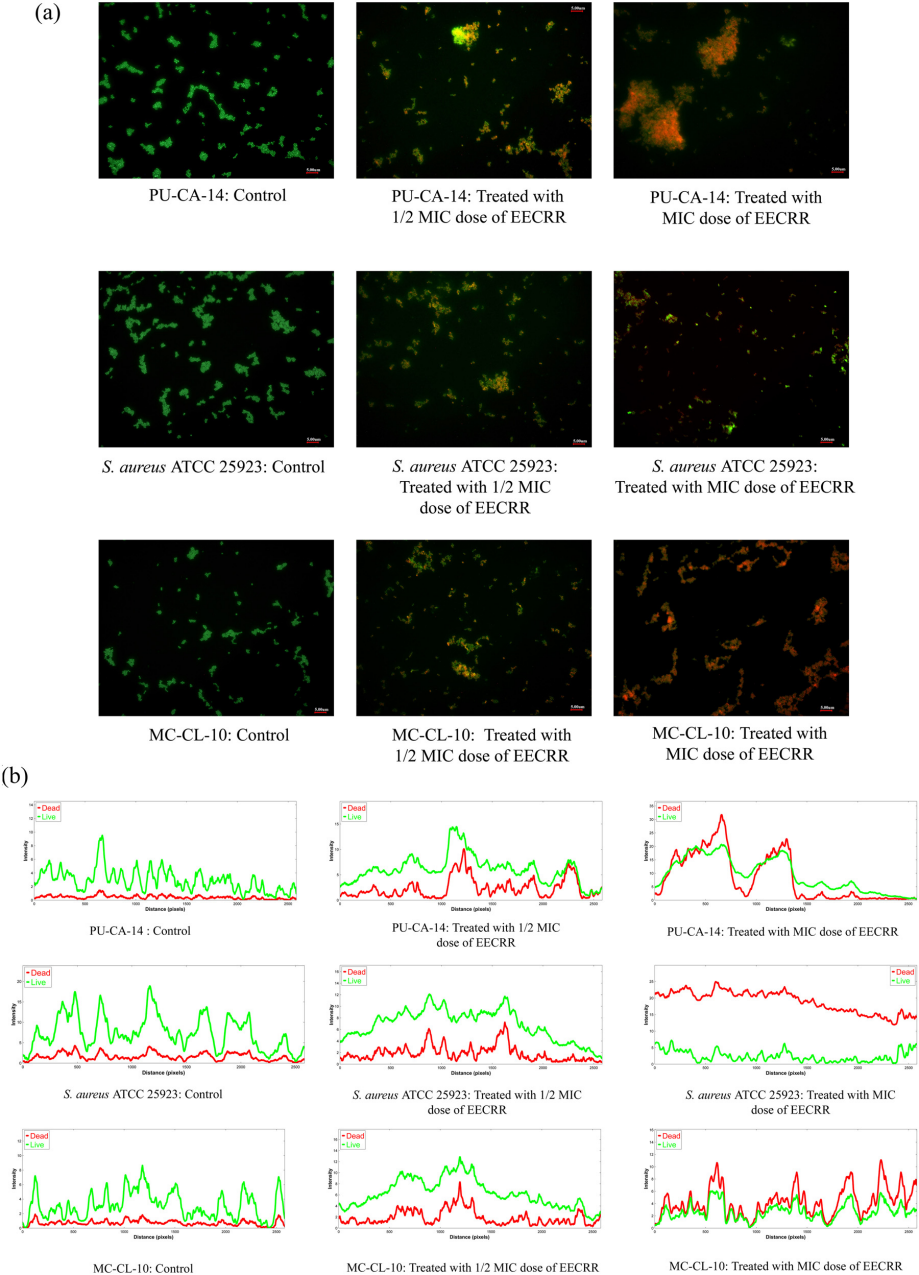
Impact of ampicillin and AO staining on EECRR-treated bacterial cells. (a) Control and EECRR-treated PU-CA-14 and *S. aureus* ATCC 25923 cells were stained with AO (scale bar = 5.0 µm), and (b) graphs show the RGB plot profiles for treated bacteria with different doses of EECRR based on fluorescence intensity profiles on merged images.

While observing bacterial cells stained with AO under a fluorescence microscope, we found that control cells fluoresce green, whereas treated cells appeared red, as AO gives green fluorescence when it intercalates with double-stranded DNA and red fluorescence after electrostatic interaction with single-stranded DNA or RNA. All control merged images (Figure 2a) represent viable cells that are depicted as green bacterial cells. Additionally, the red green blue (RGB) plot profile analysis of the control images (Figure 2b) demonstrates a substantial degree of overlapping of the green fluorescence peaks over the red fluorescence peaks, providing further evidence that the bacterial cells observed in the control images are viable. Likewise, the intensity of red fluorescence peaks overlaps the green fluorescence peaks, indicating dead bacterial cells on exposure to MIC dosage of EECRR.

### 3.3 Impact of EECRR on plasmid band pattern of bacterial cells

**Figure 3:**
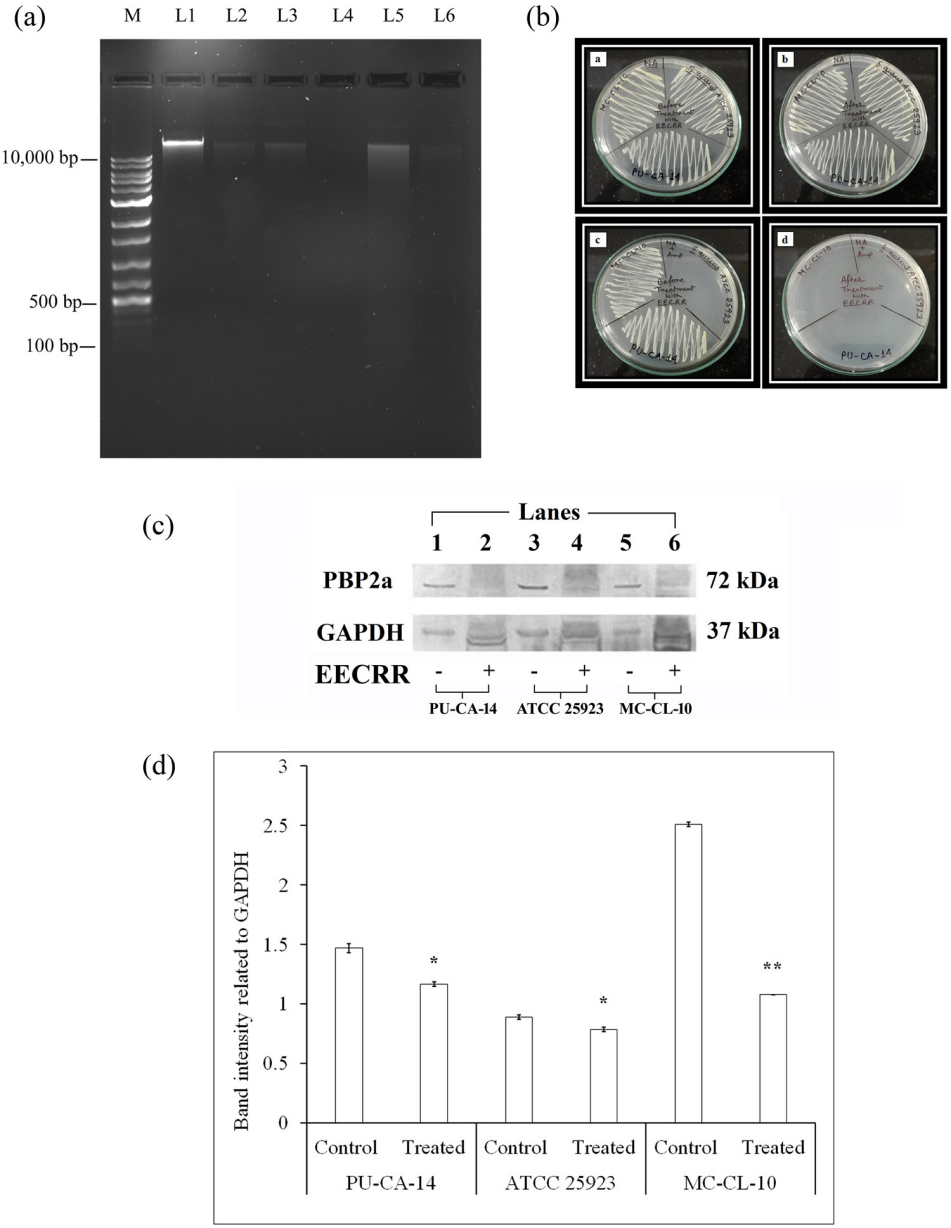
(a) Impact of EECRR on plasmid band integrity. Prominent plasmid bands are visible in control samples of PU-CA-14 (L1), ATCC 25923 (L3), and MC-CL-10 (L5) when compared to the EECRR-treated samples of PU-CA-14 (L2), ATCC 25923 (L4), and MC-CL-10 (L6). (b) Inhibition of the growth of MAR-SA on treatment with EECRR in the presence of ampicillin. Growth of control bacterial cells on NA plate without ampicillin (a) and with ampicillin (c), growth of EECRR-treated bacterial cells on NA plate without ampicillin (b) and with ampicillin (d). (c) Expressions of PBP2a in control and EECRR-treated cells of PU-CA-14, *S. aureus* ATCC 25923, and MC-CL-10 analysed by immunoblotting. PBP2a expressions decreased in EECRR-treated samples of PU-CA-14 (lane 2), *S. aureus* ATCC 25923 (lane 4), and MC-CL-10 (lane 6) compared to control samples (lanes 1, 3, and 5, respectively). GAPDH was used as the loading control in this study. (d) Densitometric analysis of immunoblots to quantitate PBP2a expression in control and EECRR-treated cells of PU-CA-14, *S. aureus* ATCC 25923, and MC-CL-10. The PBP2a protein levels were quantified by analysing the band intensity of each PBP2a and GAPDH band using ImageJ software. PBP2a levels were normalized to GAPDH protein levels and used as a control of total protein loading. The significance level is based on Mann–Whitney *U* multiple comparison test (*p < 0.05 and **p < 0.01). Data are presented as mean ± SE, *n* = 3.

After extracting plasmid DNA from EECRR-treated cells, we observed the presence of band (>10,000 bp) in all control cells, whereas in treated cells (PU-CA-14 and MC-CL-10), bands were blurred and no visible band was noted in *S. aureus* ATCC 25923 after EECRR treatment (Figure 3a).

### 3.4 Growth of EECRR-treated bacterial cells in the presence of antibiotics

The growth of bacterial cells on NA plates was checked in the presence and absence of antibiotics by the replica-plating method. The results of this experiment revealed that the cells were unable to grow on NA plates with antibiotic (ampicillin) after treatment with EECRR (Figure 3b).

### 3.5 Influence of EECRR on the expression of PBP2a protein

Western blot technique was applied to analyse the expression of PBP2a protein in control and EECRR-treated *S. aureus* cells (PU-CA-14, *S. aureus* ATCC 25923, and MC-CL-10) by anti-PBP2a antibody. Treated bacterial cells were exposed to a 1/2 MIC dose of EECRR. The expressions of PBP2a (molecular mass: 72 kDa) were remarkably low (*p < 0.05 and **p < 0.01) in all treated cells when compared to the control cells that exhibited prominent bands of PBP2a (Figure 3c and d). The difference between the expressions of PBP2a is maximum in control and treated cells of MC-CL-10 (*p* < 0.01).

### 3.6 Impact of EECRR on DNA integrity

**Figure 4:**
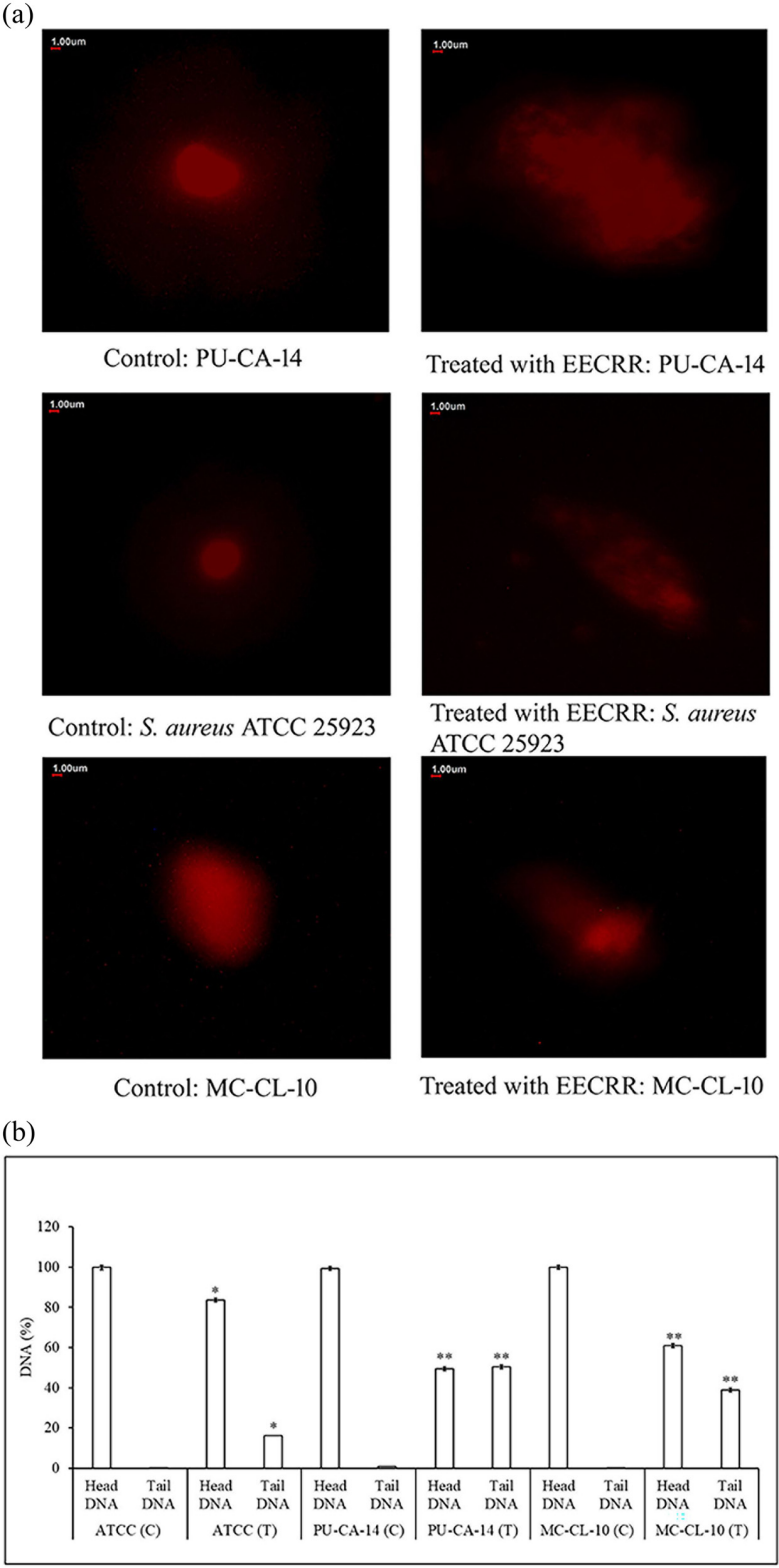
Effect of EECRR on genomic DNA of *S. aureus* by (a) comet assay (scale bar = 1.0 μm) and (b) its characteristic analysis. The length of the head and tail of DNA was altered in control (C) and treated (T) bacterial cells. The degree of damage was represented as the percentage of the lengths of the head and tail of comets formed with the help of the CASP software. The significance level is based on Mann–Whitney *U* multiple comparison test (*p < 0.05 and **p < 0.01).

Detection of damaged DNA double strands by comet assay was studied, and it was found that under constant electrophoretic conditions, the distance migrated by severely damaged DNA is longer compared to that by intact DNA. Data obtained from the comet assay by treatment with EECRR at concentrations of 384.8, 96.8, and 392.8 µg/mL are shown in Figure 4. The length of the DNA tail was significantly longer in all three types of *S. aureus* cells treated with EECRR in the form of a “comet” compared to the control sets.

## 4 Discussion

The skin and mucous membranes of 30% of the human population serve as the natural habitat for microbiota like *S. aureus* [25]. *S. aureus* has been detected as a causative agent for ailments, ranging from mere skin infection to severe life-threatening diseases, such as endocarditis, pneumonia, food poisoning, septicaemia, septic shock, post-transplantation liver infection, UTI, and end-stage kidney failure in haemodialysis patients [2,12]. The emergence of MRSA strains has made the management of these diseases more complicated.

The severity of infections can be determined by the physical state of patients since elderly patients are more prone to *S. aureus* self-infection owing to various other types of age-related illnesses [26]. The health status of elderly individuals may not allow them to cope with the adverse effects caused by the application of new drugs with different chemical configurations. The present study is a follow-up to our previous investigation based on the antibacterial, anti-inflammatory, and hepatoprotective role of *C*. *roseus*. The composition of the plant extract has already been discussed in our previous studies. To highlight a few findings from those studies, EECRR contains two fatty acid esters, 9,12-octadecadienoic acid (*Z,Z*)-2-hydroxy-1-(hydroxymethyl) ethyl ester and 9,12-octadecadienoyl chloride, an essential oil, namely, 1-nonadecene and pentacyclic triterpenoid called ursolic acid, as assessed by gas chromatography–mass spectrometry [13] and high-performance liquid chromatography [14]. Existing literature provides evidence that these bioactive compounds are found in EECRR and are effective against the growth of bacteria. Essential oils and fatty acids mainly have membrane-damaging effects on bacterial cells. In our previous study, we have shown that EECRR causes the inhibition of efflux pump activity, which helps the antibacterial agents to exert their maximum effects without being flushed out from the cells and also generates intracellular reactive oxygen species (ROS), which kills bacteria by damaging DNA [14]. Moreover, ursolic acid, being one of the active phyto-components present in EECRR, can have such DNA-damaging properties as stated in the study of Messner et al. [27].

In this study, AO staining of *S. aureus* shows that a high dose of EECRR (MIC dose) can kill more bacterial cells as indicated by high intensities of red fluorescence, possibly due to DNA damage that results in the accumulation of ssDNA. A comet assay is a sensitive approach developed recently to quantify DNA damage at the single-cell level and identifies genotoxic substances [28]. Prominent DNA damage, observed by the comet assay, validates the cause of cell death on treatment with MIC doses of EECRR. It is already evident that the generation of ROS is one of the reasons for damaging DNA [29] and EECRR has the potency to produce a huge amount of ROS within the bacterial cells [14]. However, the amount of ROS generated by EECRR in mammalian cells is not that significant to cause damage to eukaryotic cells, as observed in the experimental rat model of our earlier investigation [14]. The extract may also influence the other structures of the bacterial cells, as we have previously reported, and it damages the cell envelope as assessed by scanning electron microscopy [13], inactivates efflux pumps present on bacterial cell membranes, and generates ROS within bacterial cells [14]. Our current finding is that the bacterial DNA-damaging capability of EECRR is another key attribute and is essential to combat MAR-SA infection.

Exposure of *S. aureus* cells to low doses of EECRR results in low intensities of red fluorescence in comparison with that of MIC dose, indicating a lesser number of dead cells. The remaining live cells were subjected to plasmid isolation to investigate whether there was any alteration in plasmid DNA. Our observation was in favour of the results that we obtained after AO staining. This finding persuades us to further explore the mechanism of the loss of antibiotic resistance genes, as in most cases, they reside on the bacterial plasmid. Reported literature suggests that plant extracts have the potential to suppress the antibiotic resistance gene by eliminating or curing plasmids [30,31] and reducing the antibiotic resistance capacity of MRSA [32]. It is also documented that the plasmid-curing effects of the herbal extract can be visualized in gel images, which is analogous to our study [33].

The satisfactory outcome of the replica-plating experiment demonstrates that the underlying mechanism is perhaps due to plasmid curing, as the cells after treatment with EECRR were unable to grow on ampicillin-containing plates. Relevant observation is mentioned by other researchers in this regard [21].

To achieve conclusive results, we targeted the PBP2a expression level as a precise indicator of beta-lactam antibiotic resistance. Resistance to the β-lactam antibiotics, including ampicillin, penicillin, and oxacillin, is principally mediated by the production of PBP2a, encoded by the *mecA* gene [34]. Čuvalová et al. also detected the presence of the *mecA* gene in multiresistant staphylococcal species producing PBP2a [35]. According to our experiment, the expression of PBP2a was downregulated in EECRR-treated bacterial cells. Reduced expression of the same protein was also observed in an experiment where MRSA was treated with phlorofucofuroeckol-A [36]. The unavailability or reduced synthesis of PBP2a, due to the effects induced by EECRR on *S. aureus*, is likely to be the key determinant for the decline in antibiotic resistance observed in MAR-SA strains.

**Figure 5:**
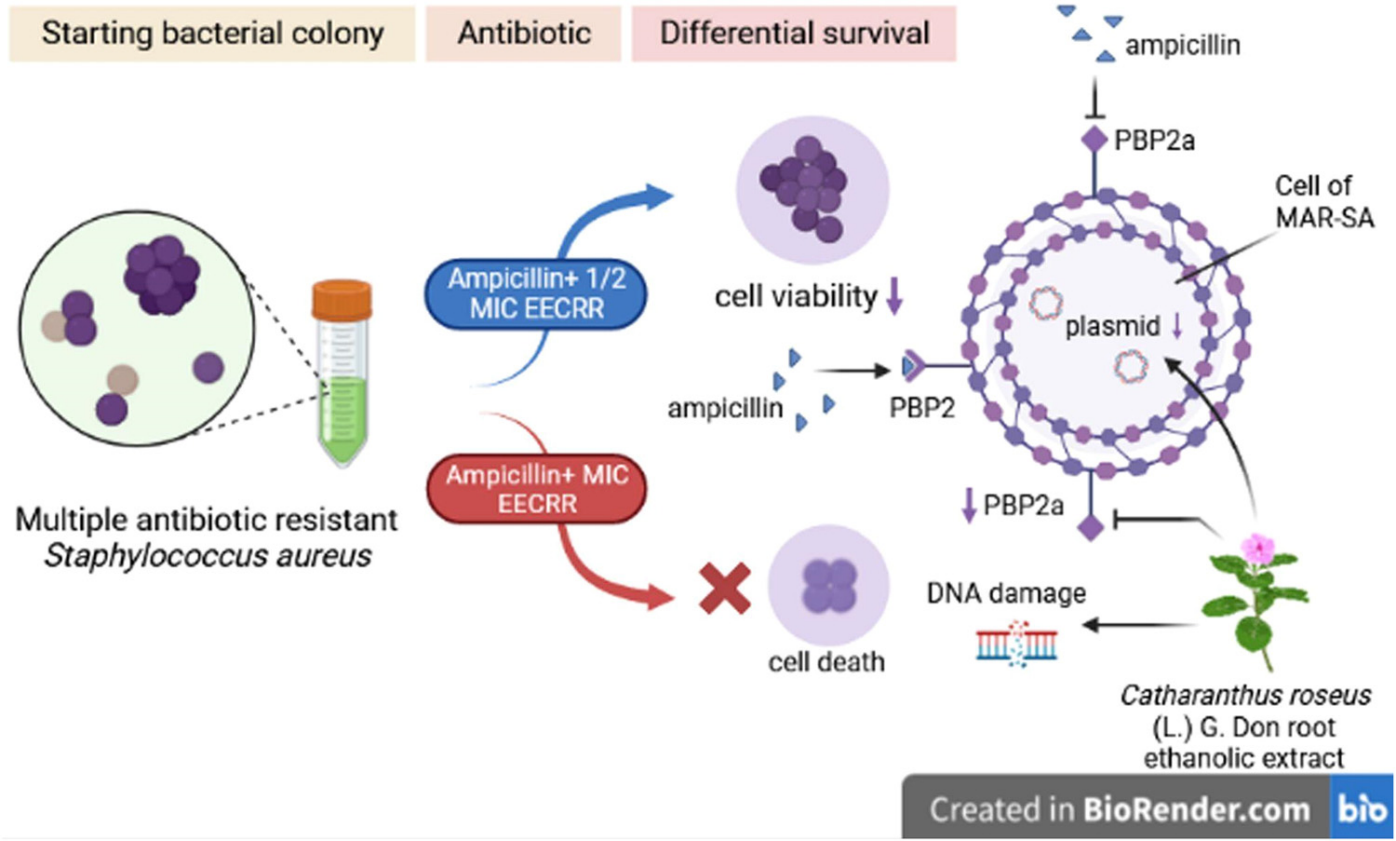
Summary of the current study. EECRR has the potential to enhance the sensitivity of ampicillin-resistant *S. aureus* strains to ampicillin by decreasing the levels of PBP2a protein, which promotes antibiotic resistance. The decrease in PBP2a expression results in a negative impact on the bacteria’s ability to resist antibiotics, as it disrupts the integrity of their plasmid DNA.

Moreover, eight- and four-fold reduction in MIC values of ampicillin was observed in *S. aureus* strains of PU-CA-14 and MC-CL-10, respectively, after treatment with EECRR, and increased diameter of zone of bacterial growth inhibition in disc diffusion assay supports the finding that ampicillin-resistant bacterial cells became quite sensitive to β-lactam antibiotics. This outcome is supported by the works of other investigators also [37]. This implies that the extract has inactivated the normal activity of bacterial cells and made them incapable to harm the host. Therefore, EECRR has the potential to increase the susceptibility of MAR-SA to ampicillin. Figure 5 presents a schematic diagram elucidating the EECRR's potential role in augmenting the sensitivity of ampicillin-resistant *S. aureus*.

## 5 Conclusion

This study shows that a high dose of EECRR (at MIC value) was capable of killing *S. aureus* cells by causing DNA damage. Additionally, at a low dose (1/2 MIC value), EECRR can reduce the expression of the PBP2a protein and affect the plasmid DNA of *S. aureus*. The plasmid in bacterial cells carries genes responsible for antibiotic resistance, which led to a change in the ARP in *S. aureus* strains. Consequently, *S. aureus* strains, previously resistant to ampicillin, have become partially sensitive to it due to the effects of EECRR. Additional experiments are required to enhance the susceptibility of bacteria, transitioning them from partially susceptible to completely susceptible to the combined effect of the plant extract and antibiotic. Nevertheless, this achievement is beneficial as it will allow previously ineffective antibiotics to regain the effectiveness, thereby simplifying the treatment of infectious diseases caused by multidrug-resistant bacterial strains. Moreover, this treatment approach, after EECRR passes the safety assessment to conduct clinical trials, would be cost-effective as affordable antibiotics like ampicillin can be reused again for therapeutic purposes.
